# OB-Folds and Genome Maintenance: Targeting Protein–DNA Interactions for Cancer Therapy

**DOI:** 10.3390/cancers13133346

**Published:** 2021-07-03

**Authors:** Sui Par, Sofia Vaides, Pamela S. VanderVere-Carozza, Katherine S. Pawelczak, Jason Stewart, John J. Turchi

**Affiliations:** 1 Indiana University Comprehensive Cancer Center, Indiana University School of Medicine, Indianapolis, IN 46202, USA; spar@iu.edu (S.P.); sviades@iu.edu (S.V.); 2 Department of Medicine, Indiana University School of Medicine, Indianapolis, IN 46202, USA; vandervp@iu.edu; 3 NERx Biosciences, Indianapolis, IN 46202, USA; kspawelczak@nerxbiosciences.com; 4 Department of Biological Sciences, University of South Carolina, Columbia, SC 29208, USA; JASTEWAR@mailbox.sc.edu; 5 Department of Biochemistry and Molecular Biology, Indiana University School of Medicine, Indianapolis, IN 46202, USA

**Keywords:** DNA binding, OB-fold, genome stability, single-stranded DNA, DNA damage response, DNA repair, DNA replication, cancer therapy, drug development

## Abstract

**Simple Summary:**

Genome replication, repair, recombination, and the DNA damage response (DDR) rely on a cadre of DNA binding proteins to execute and regulate these critical pathways. There are many structural motifs that support DNA-binding and included in these is the oligonucleotide/oligosaccharide binding fold (OB-fold). OB-folds are found across the tree of life and play critical roles in chromosome maintenance and stability. This paper reviews the current state of knowledge regarding structure, function, and involvement of OB-fold proteins in the DNA damage response, replication, and repair. We highlight a group of OB-fold DNA binding proteins and discuss how disruption of these critical protein–DNA interactions can be exploited in cancer therapy.

**Abstract:**

Genome stability and maintenance pathways along with their requisite proteins are critical for the accurate duplication of genetic material, mutation avoidance, and suppression of human diseases including cancer. Many of these proteins participate in these pathways by binding directly to DNA, and a subset employ oligonucleotide/oligosaccharide binding folds (OB-fold) to facilitate the protein–DNA interactions. OB-fold motifs allow for sequence independent binding to single-stranded DNA (ssDNA) and can serve to position specific proteins at specific DNA structures and then, via protein–protein interaction motifs, assemble the machinery to catalyze the replication, repair, or recombination of DNA. This review provides an overview of the OB-fold structural organization of some of the most relevant OB-fold containing proteins for oncology and drug discovery. We discuss their individual roles in DNA metabolism, progress toward drugging these motifs and their utility as potential cancer therapeutics. While protein–DNA interactions were initially thought to be undruggable, recent reports of success with molecules targeting OB-fold containing proteins suggest otherwise. The potential for the development of agents targeting OB-folds is in its infancy, but if successful, would expand the opportunities to impinge on genome stability and maintenance pathways for more effective cancer treatment.

## 1. Oligonucleotide/Oligosaccharide Binding-Fold (OB-Folds)

**Figure 1:**
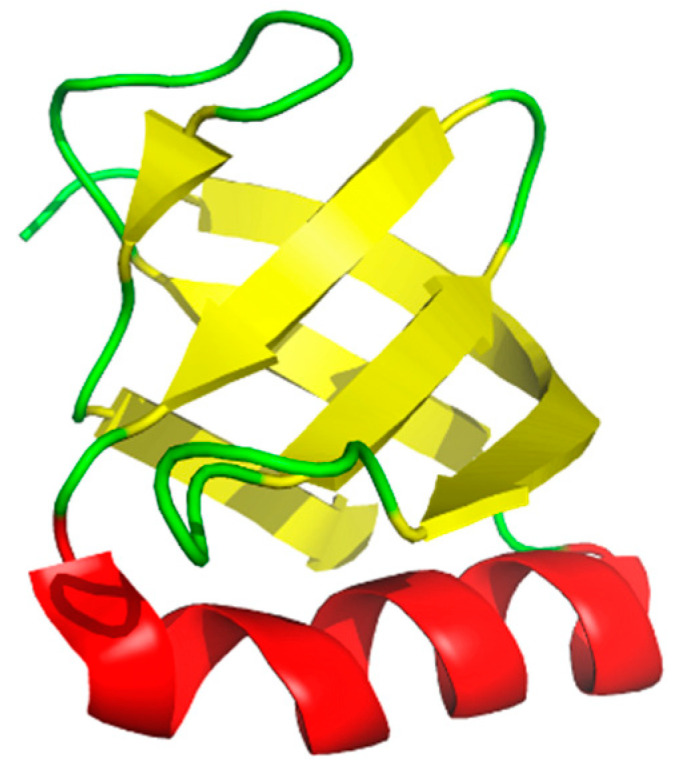
Structure of the verotoxin-1 OB-fold. Rendered from PDB:2XSC using pymol.

The OB-fold was originally described by Murzin as a common motif in a small series of proteins known to bind oligonucleotides or oligosaccharides [1]. The fold is characterized by a 5-strand beta sheet oriented to form a β-barrel (Figure 1), and the β-barrel is capped by an α-helix. The common structural features allow interactions with differing oligomers based largely on structure and chemistry. While the differing classes of OB-folds often lack sequence homology, the flexible nature of the motif allows for an ideal binding surface for protein–nucleotide interactions and similarly protein–protein interactions. Oligomer binding by these proteins is supported by the β-barrel depicted in yellow and the loop regions depicted in green (Figure 1). OB-fold proteins often contain tandem copies of the fold to allow binding of longer oligomers, especially in the case of binding DNA for replication, repair, and recombination [2,3]. In the subsequent 30 years since their discovery, a growing number of OB-fold containing proteins have been identified in archaea, bacteria, and eukaryotes as well as viruses [4]. OB-fold containing proteins participate in a multitude of biochemical pathways including DNA replication, DNA repair, cell cycle regulation, and maintenance of telomeres. In addition, mutations in OB-fold proteins can lead to a variety of human diseases [5] and development of small molecule inhibitors will be useful tools to elucidate function and interrogate these diseases. Some of the functions are essential and others participate in redundant or overlapping pathways thus the efficacy and potential toxicity of inhibitors to these proteins will likely be specific for each protein. A comprehensive understanding of these protein complexes will provide an opportunity to further characterize these various human diseases associated with mutations in OB-fold proteins and potentially aid in the identification and development of corresponding therapeutic options.

## 2. Replication Protein A (RPA)

Replication protein A is a eukaryotic single-stranded DNA binding protein that plays an essential role in DNA metabolic pathways including replication, repair, and recombination as well as activating the DNA damage response (DDR) and cell cycle checkpoints. RPA is abundant in cells and it binds to specific ssDNA intermediates in nearly all DNA maintenance pathways including nucleotide excision repair (NER), DNA mismatch repair (MMR), homologous recombination repair (HRR), and base excision repair (BER) as well as replication fork restart [6,7,8,9,10].

**Figure 2:**
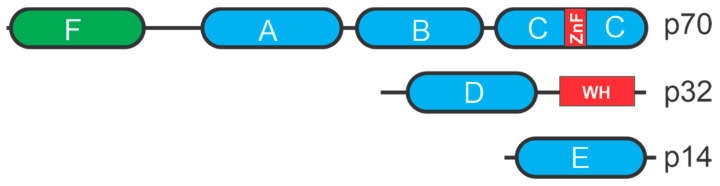
RPA structural motifs. OB-folds are depicted as ovals with other motifs depicted as rectangles. ZnF is a zing finger motif and WH a winged-helix motif.

RPA has a heterotrimeric complex with three subunits; RPA70, RPA32, and RPA14. Each subunit contains OB-fold DNA-binding domains (DBD) [11] (Figure 2). RPA70 contains four OB folds in DBD-A, DBD-B, DBD-C, and DBD-F whereas RPA32 contains an OB-fold in DBD-D and RPA14 contains an OB-fold in DBD-E [12]. RPA binds to ssDNA with high affinity that is largely dependent on the two central DBD-A and DBD-B folds in RPA70. Binding is dependent on the length of the ssDNA and shorter stretches of ssDNA results in lower binding affinity compared to longer ones suggesting that engagement of the other OB-folds can enhance binding and stability of the complex [13].

The N-terminal region of RPA70 contains an OB-fold termed DBD-F. This OB-fold possesses a basic cleft that allows for interaction with both ssDNA and specific proteins involved in DNA metabolism and the DDR [11]. The main function of this OB-fold is not in direct DNA binding but rather to facilitate interaction with other proteins. A key protein–protein interaction includes interaction between DBD-F and phosphorylated RPA32. This prevents an undesirable interaction of the phosphorylation domain of RPA32 with the main DNA-binding domain of RPA, DBD-A, and DBD-B [11]. The N-terminus of RPA70 also interacts with various DDR and DNA repair proteins such as Rad9, ATRIP, MRE11, and P53. These proteins possess an acidic alpha helical domain that allows them to bind to the N-terminal basic cleft of the DBD-F in RPA70 [14].

Convincing evidence from structural and biophysical studies has dramatically increased our understanding of how RPA binds DNA. Four critical ssDNA binding domains containing the distinctive OB folds in RPA70 and RPA32 are responsible for interacting with DNA in a dynamic fashion. Importantly, these folds are connected by mobile loops that make it a flexible complex, allowing the protein to adopt multiple conformations [15] and account for the differing roles of RPA in multiple cellular pathways. This is further emphasized by a recent FRET-based study showing that RPA-DNA binding is highly dynamic and involves three distinct binding modes that are dependent on RPA concentration and ssDNA length [16]. This study supports earlier work suggesting that RPA adopts various binding modes and conformations on ssDNA [17] and can rapidly spread along ssDNA to support the cellular response to replication stress. The flexible binding modes of RPA allow for several distinct functions that ensure a cell appropriately responds to DNA damage and replication stress [18].

The development of small molecule inhibitors of RPA has been pursued by multiple groups including ours. RPA inhibitors have been pursued on two fronts, those that block the N-terminal OB-fold and its interaction with key DDR proteins and those that disrupt RPA’s ability to bind DNA. The advantage of chemical inhibition over genetic methods to reduce RPA lie in the specificity and selectivity of chemical inhibitors that can block one function, while allowing other function to persist. A series of N-terminal OB-fold targeted inhibitors have been identified using different screening methodologies, fragment-based NMR screening, and a biochemical high-throughput screen [14,19,20,21]. Both classes of inhibitors bind reversibly and serve to block protein–protein interactions. Analysis of the cellular effects of RPA inhibition demonstrated that RPA inhibitors blocked DNA damage dependent phosphorylation of RPA, resulting in increased DNA replication stress in cancer cells but not in normal cells [22]. Importantly, these protein–protein interaction inhibitors targeting the N-terminal fold do not inhibit ssDNA binding activity.

Work in our lab has focused on small molecule inhibitors of the RPA–DNA interaction, identifying inhibitors that bind to the OB-folds of DBD-A and -B in the central region of RPA70 [23,24]. Reversible inhibitors were initially identified and shown to possess in vitro and cellular activity [25] with later derivatives optimized for potency [26], cellular uptake, and in vivo activity [27,28]. RPA inhibitor mechanism of action studies suggest a role in the DDR as opposed to S-phase replication, nucleotide excision repair, or homologous recombination. Consistent with models of RPA exhaustion [29,30] where the single stranded DNA exceed the capacity of RPA binding activity, selective inhibition of RPA’s single strand binding activity may induce a state of chemical RPA exhaustion that should prove effective to illicit single agent anticancer activity and synergize with select DDR inhibitors and traditional chemotherapeutics that induce DNA damage [28].

## 3. CST

**Figure 3:**
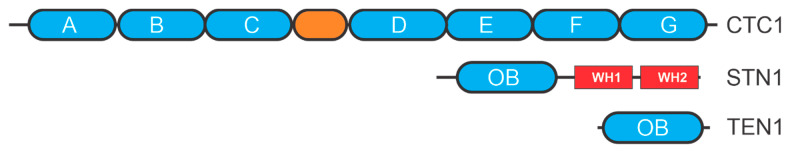
CST structural motifs. OB-folds are depicted as ovals with other motifs depicted as rectangles. WH is a winged-helix motif.

Like RPA, CST is a heterotrimeric single-stranded DNA binding protein that plays a primary role in protecting chromosome ends and in telomere length regulation. In vertebrates and plants, CST is named for its component three proteins, CTC1, STN1, and TEN1 whereas in *S. cerevisiae*, where it was first discovered, it is composed of Cdc13, Stn1, and Ten1 [31,32,33,34,35]. CTC1 and Cdc13 both contain multiple OB-folds (four in Cdc13 and seven in CTC1), but CTC1 appears to have evolved from RPA versus Cdc13, as the OB-folds of CTC1 more closely resemble those in RPA70 than Cdc13 [36] (Figure 3). STN1 and TEN1, on the other hand, are structurally conserved across organisms [36,37]. Both contain a single OB-fold that is structurally similar to RPA32 for STN1 and RPA14 for TEN1. STN1 also contains two winged-helix domains in comparison to a single winged-helix domain in RPA32. In humans, purified CST can form a multimeric structure, composed of ten separate CST complexes [36]. The physiological relevance of this higher order structure is still to be determined.

CST uses its multiple OB-folds to increase DNA binding affinity akin to RPA [38,39]. By engaging multiple OB-folds, dissociation of a single fold does not necessarily lead to dissociation from the DNA. This creates a tighter binding affinity, decreased off rates, and provides flexibility for dynamic binding to a variety of DNA substrates. Work is still ongoing to characterize the OB-folds of mammalian CST including determining which ones are necessary for stable binding. However, data suggest that the OB-folds in both the N- and C-terminal domains of CTC1 and the OB-fold in STN1 bind to ssDNA [32,36,39]. In terms of sequence specificity, CST has both specific and non-specific binding modes, a feature that may be amenable to drug targeting. On short stretches of ssDNA (18 nt), CST shows a strong preference for G-rich sequences such as the telomeric G-strand [40]. As the DNA length increases, this sequence specificity diminishes, with no preference observed between G-rich and random sequences on ssDNA of 36 nt or longer. The binding footprint of CST has not been determined but maximum binding affinity (K_D_ = 0.3 nM) was observed on a 48 nt ssDNA sequence [40]. In addition to ssDNA, CST has preference for ss-dsDNA junctions [39]. Another feature of CST is its ability to unfold DNA secondary structures called G-quadruplexes (G4s) [39,41,42]. G4s consist of four repeats of at least three guanosines to form a four stranded structure through Hoogsten base pairing [43]. These structures are highly abundant in the genome and play both positive and negative roles in transcription and DNA replication. The dynamic nature of OB-folds and shear number of repeat motifs in CST are likely to facilitate modulation of the G4 structures.

While the architecture may differ between organisms, CST plays a primary role in telomere maintenance and stimulates DNA polymerase α-primase (pol α) in all organisms [44]. In *S. cerevisiae,* CST functions in chromosome end protection as well as regulating telomere length. In vertebrates, the shelterin complex is primarily responsible for end protection, but recent work suggests that CST may also function in end protection and the DDR at telomeres [45,46]. Generally, CST is involved in telomere length regulation where it terminates telomerase activity to prevent over-extension of the G-rich ssDNA overhang (G-overhang) and stimulates pol α activity to convert the overhang, generated by telomerase, back to duplex DNA, a process known as C-strand fill-in. While this switch between telomere extension and C-strand fill-in is not fully characterized in mammals, it is coordinated by TPP1, in place of Cdc13, and involves the recruitment of CST to terminate telomerase activity and stimulate C-strand fill-in [47,48,49]. We suspect that post-translational modification will play an equally important role in mammals as it does in yeast.

In mammals, CST is also involved in telomere duplex replication, where it is proposed to remove, or prevent, G4s that impede replication through the G-rich telomeric DNA [50,51]. Furthermore, increasing evidence indicates that CST acts at non-telomeric sites to promote DNA replication and repair [52]. At stalled replication forks, CST has been proposed to load RAD51 for fork restart, prevent MRE11 cleavage of regressed forks, activate dormant replication origins, and promote chromosome cohesion [51,53,54]. These roles in DNA replication rescue may involve G4 unfolding, since CST primarily localizes to G-rich sequences following hydroxyurea-induced fork stalling [54]. Binding to G4s could facilitate the localization of CST to specific regions such as telomeres and other G-rich DNA sequences. However, CST also interacts with the replisome components, MCM2-7, AND-1/Ctf4, and pol α, which may localize it to, or stabilize interaction with, stalled forks [55]. Moreover, depletion of STN1 or CST overexpression increases or decreases MCM2-7 loading in G1, suggesting that CST negatively affects origin licensing [55]. Although the consequences of over-licensing in the absence of CST are still not well understood, CST may help to properly distribute replication origins to ensure complete genome duplication. Finally, CST was recently identified in several loss-of-function screens for PARP inhibitor resistance in BRCA-deficient cancer lines and subsequently shown to promote non-homologous end joining by facilitating pol α-dependent fill-in of resected DSBs [56,57,58], making this an intriguing novel target for cancer therapy.

Based on its varied functions in telomere biology and DNA replication/repair, depletion of human CST leads to genome instability in the form anaphase bridges, micronuclei, telomere dysfunction, and chromosome fragility [31,51,54]. Deletion of CTC1 in human cells and mice induces G2/M arrest, cellular senescence, and apoptosis [59]. While CTC1 deletion was not embryonic lethal in mice, they had a median lifespan of only 24 days, often dying from bone marrow failure caused by the loss of hematopoietic stem cells. Interestingly, conditional deletion of human TEN1 has much less severe phenotypes than CTC1 deletion including an absence of growth defects, telomere elongation, and telomeric DNA damage signaling [47]. Mechanistically, Feng et al. determined that this was due to the ability of CTC1-STN1 to maintain inhibition of telomerase in the absence of TEN1, thus preventing hyper-elongation of G-overhangs and RPA binding. There are currently no reports on mammalian STN1 deletion; however, it is predicted that the phenotypes will be similar to deletion of CTC1, based on previous reports that CTC1 stability is dependent on STN1 [60]. In addition to the effects of CST deletion or depletion on genome instability and cell growth, depletion of STN1 increased the sensitivity of cancer cells to various replication inhibitors that stall DNA polymerases [61]. Conversely, overexpression of CST increased cell survival following treatment with the same inhibitors. Overexpression of STN1 and TEN1 also stimulated senescence bypass, a strategic action that could relate to carcinogenesis [62].

Mutations in CTC1 and STN1 are associated with two congenital genetic disorders, namely Coats plus (CP) syndrome (also known as cerebroretinal microangiopathy with calcifications and cysts) and dyskeratosis congenita (DKC) [63]. While DKC is caused by mutations in several telomere maintenance proteins, genetic alterations in CP are almost exclusively in CTC1 or STN1 [64]. Single nucleotide polymorphisms in CTC1 and STN1 are correlated with an increased risk of various cancers [65,66,67,68,69,70]. Decreased expression of CST subunits also correlates with decreased survival in breast, lung, and gastric cancer patients [71]. Furthermore, loss of CST leads to PARP inhibitor resistance in BRCA-deficient breast and ovarian cancer lines, as above-mentioned. In contrast, increased expression of CTC1 increased radioresistance in patient-derived melanoma cells by preventing telomere shortening and apoptosis [72].

Despite the correlation of CST dysfunction with cancer, the precise role(s) of CST in cancer development and progression is still not clear. However, due to its defined roles in telomere biology and DNA replication/repair and similarities to RPA, CST may be a promising target for cancer therapy. This may be particularly true due to its primary role in telomere maintenance, a hallmark of cancer [73]. Loss of CTC1 leads to telomere DNA damage signaling due to hyper-extension of G-overhangs and RPA binding [74]. Furthermore, treatment of CTC1 deleted cells with the ATR inhibitor VE-821 significantly increased apoptosis [46]. Therefore, targeting the OB-folds in CST to induce telomere dysfunction/genome instability along with DDR inhibitors may be an effective cancer therapy.

## 4. hSSB1/2

**Figure 4:**
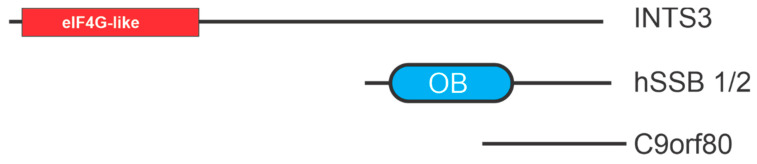
hSSB structural motifs. OB-folds are depicted as ovals with other motifs depicted as rectangles. eiF4G is a motif found in eukaryotic initiation factor 4G.

Human single-stranded binding proteins 1 and 2, termed hSSB1 and hSSB2, were first identified in 2008 [75]. Data demonstrate that hSSB1 is involved in HR while hSSB2 is involved in the recognition of bulky DNA damage linking both to the DNA damage response [76,77,78]. Both proteins share a similar composition including a single N-terminal OB-fold and short C terminal disordered region, and both are highly conserved among the metazoans, showing a more similar composition to the bacterial SSB then RPA. The hSSB1 protein differs from hSSB2 in function. hSSB1 binds to ssDNA substrates and occludes of a region 5–6 nt in agarose gel shift assay, while there was no binding activity reported for hSSB2. Similarities between RPA and hSSB1 include an almost equivalent binding affinity to ssDNA. Unlike RPA, hSBB1 may have a greater area of functioning, in which it binds to short duplex DNA constructs (33 nt) as well as duplex DNA with short overhangs (6 nt) [8]. hSSB1 and hSSB2 can each interact with an integrator complex subunit 3 (INTS3) and C90ORF80 to form trimeric SS binding complexes termed SOSS1/2 (sensors of single stranded DNA) complexes [79] (Figure 4).

In addition to complexation with the INTS3 and C9orf80, hSSB1 forms protein–protein interactions with NBS1, a component of the MRE11-RAD50-NBS1 (MRN) repair complex that was recently shown to be regulated by SUMOylation [80,81]. Depletion of hSSB1 inhibited MRN recruitment to sites of DSB and diminishes MRN nuclease activity. The OB-fold domain of hSBB1 has a poly(ADP ribose) (PAR) binding domain, and the interaction between PAR and hSSB1 is crucial to the early engagement of hSSB1 at sites of DNA damage [82]. Depletion of hSSB1 diminishes ATM kinase activity as well as increases radiosensitivity, causes defects in G1/S and G2/M checkpoint activation, and enhances genomic instability. Protein–protein interactions have been identified between hSSB1 and protein p21, a potent cyclin-dependent kinase inhibitor. This interaction prevents ubiquitin-mediated degradation of p21. Through these interactions, hSSB1 stabilizes p21 and strengthens the G1/S transition and G2/M checkpoints. hSSB1 also has crucial actions in the p53 pathway. In this capacity, hSSB1 stabilizes p53 and interacts with the acetyltransferase p300 to promote p53 acetylation at Lys382, which in turn enhances p21 expression [8].

Studies in adult mice support the characterization of SSB1 and its function in genomic stability, cell cycle checkpoints, and radioresistance. After deletion of SSB1, adult mouse cells had increased radiosensitivity and IR-induced chromosome breaks. Mice also presented cancer susceptibility and had a vast number of tumors including splenic and metastatic B lymphomas, T cell lymphoma in the thymus, hepatocellular carcinoma, and B or T lymphoblastic leukemia. Furthermore, increased acetylation of hSSB1 was observed in cells treated with DNA damaging agents and was reported to decrease tumor growth. Furthermore, the inhibition of hSSB1 acetylation through use of a p300/CBP inhibitor improved chemo and radiotherapy, making it a promising cancer therapy target when combined with these treatments [8].

The role of hSSB1 and 2 in genome stability and maintenance makes these attractive targets for disrupting these critical pathways for cancer therapy. OB-fold targeted agents specific for these two proteins would also be extremely useful in delineating their specific roles in maintaining genome integrity and in the DDR and replication stress. One could envision a distinct set of synthetic lethal interactions with hSSB1/2 that would differ from other DDR targeted therapeutics, which could then allow targeting of a greater degree of genetic alterations in the DDR pathway that are observed in cancer.

## 5. MCM (Minichromosome Maintenance Complex)

**Figure 5:**
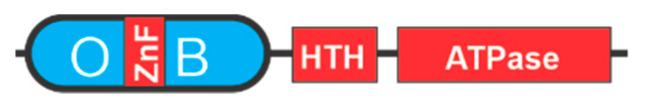
Generalized structural motifs in MCM proteins. OB-folds are depicted as ovals with other motifs depicted as rectangles. HTH is a helix-turn-helix motif. ATPase and zing finger motifs (ZnF) are as indicated.

First identified in *S. cerevisiae*, the minichromosome maintenance complex (MCM) plays a central role in DNA synthesis [83]. MCM is a heterohexamer made up of six related polypeptides MCM2-MCM7, which form a ring-link structure. MCM complexes are crucial for genomic DNA replication, elongation, RNA transcription, chromatin remodeling, and genome stability due to its ATP-dependent DNA helicase activity. Each MCM subunit consists of two domains, the N-terminal domain that facilitates DNA and protein interactions, and the C-terminal domain, which possesses the ATPase and helix-turn-helix (HTH) domains [84] (Figure 5). The N-terminal domain is mainly used for structural organization. The MCM OB-fold plays a crucial role in DNA binding and assists many MCM2-7 functions during replication initiation [85]. The C-terminus ATPase domain is essential for DNA unwinding, and is in the AAA+ class of ATPases [85].

The MCM complex unwinds the double stranded DNA at replication origins, recruiting DNA polymerases and initiating DNA synthesis [86]. During the G1 phase of the cell cycle, the MCM2-7 complex is loaded onto the DNA as an inactive double hexamer at origins of replication to form a pre-replication complex (pre-RC) [87]. The MCM2-7 is recruited and loaded on double stranded DNA by the origin recognition complex (ORC), Cdc6, and Cdt1 [84]. Due to the loading of MCM on dsDNA being an active process, ATP hydrolysis is required by both ORC1-6 and Cdc6. During helicase loading, DNA is inserted through an opening in the ring at the MCM2-MCM5 gate, and the opening is then closed and MCM encircles the DNA [84]. Upon helicase activation, MCM DNA interactions occur in a 3′ to 5′ manner on the ssDNA template. Once the pre-RC loads the MCM complex onto DNA, Orc1-6 and Cdc6 are no longer needed. In the early S phase of the cell cycle, the pre-RC is activated for DNA unwinding through the activity of cyclin-dependent kinases (CDKs) and Dbf4-dependent kinase (DDK), which also promotes the assembly of replication forks. The loading of additional MCM2-7 complexes is prevented in S-phase to ensure that there is only a single round of DNA replication.

Stabilization of the replication fork is required when problems arise during replication such as the loss of replication fork integrity or insufficient deoxyribonucleotides to prevent chromosome rearrangements and DNA double-strand breaks. To stabilize stalled replication forks, MCM2-7 interacts with Mrc1, Tof1, and Csm3. Without these proteins, dsDNA unwinding and replisome movement by MCM2-7 continues, but DNA synthesis stops [87]. Uncoupling of helicase from polymerase is part of normal replication, but it can result in replication stress that must be mitigated to maintain genome stability [88].

Due to their role in genome duplication, deregulation of MCM can result in chromosomal defects that may contribute to tumorigenesis. The MCM proteins have been shown to be highly expressed in malignant cancers cells as well as pre-cancerous cells that are undergoing malignant transformation [89]. MCM2 has been shown to be constantly expressed in rapidly growing cells of premalignant lung cancer [90]. MCM2 expression is also related to a higher mitotic index in breast cancer specimens [91]. Additionally, a series of recent studies correlated MCM expression with the development of hepatocellular carcinoma [92,93,94,95]. The MCM proteins are not highly expressed in somatic cells that have withdrawn from the cell cycle, making MCM proteins an ideal diagnostic marker for cancer and promising targets for the development of anti-cancer drugs [90,96].

## 6. BRCA2

**Figure 6:**
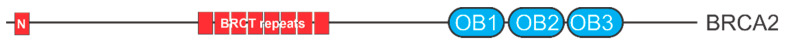
BRCA2 structural motifs. OB-folds are depicted as ovals with other motifs depicted as rectangles. BRC repeats and N- and C-terminal motifs are as indicated.

BRCA2 is a tumor suppressor with a clearly defined role in HRR, an error-free repair pathway, and a primary cellular mechanism for the repair of DSBs. In HRR, BRCA2 promotes the formation of RAD51 nucleoprotein filaments, a critical step required for strand invasion during HR mediated repair. Interestingly, BRCA2 has more recently been shown to play a role in protecting replication forks. The intricacies of its cellular roles are likely due to the large and complex nature of BRCA2. Structural characteristics include a central region with eight BRC repeats, a C-terminal motif that drives binding to Rad51, and three tandem OB-folds that are highly conserved and comprise the ssDNA binding domain. Importantly, these OB-folds are also critical for the DNA repair activity of BRCA2 and are the location of numerous cancer associated mutations [97] (Figure 6).

In the HRR pathway, BRCA2 binds PALB2 and mediates loading of RAD51 recombinase onto resected DNA ends, resulting in the formation of the RAD51-ssDNA filament. This filament prevents the involvement of the 3′-ssDNA in the damaging single-strand annealing (SSA) pathway. The helical domain and OB1 and OB2 interact with the highly conserved DSS1 protein, a critical interaction for HRR that also promotes the RAD51-loading activity of BRCA2 and mediates BRCA2 stability in cells [98]. It has also been suggested that this interaction is important for limiting the accumulation of R-loops, RNA:DNA hybrid structures. A recent study demonstrated that BRCA2 binding to DSS1 and ssDNA leads to a rearrangement of BRCA2 that stabilizes the monomeric state, and suggests that this monomeric BRCA2 in complex with DSS1 and ssDNA could be the active form in recombinational DNA repair as well as replication fork stabilization [99].

BRCA2 germline mutations significantly increase risk for prostate and pancreatic cancer and cause hereditary breast and ovarian cancer syndrome (HBOC) [100]. Unlike BRCA1 mutations typically found in basal-like cancers, BRCA2 breast cancers are generally of the luminal subtype (hormone-receptor positive), and as such are predominantly estrogen-receptor positive. BRCA2 truncation and point mutations have also been identified, and biallelic mutations and were identified as drivers of Fanconi anemia [98]. Studies have also shown that the OB-folds drive BRCA2 recognition of DNA-damage induced PAR and lead BRCA2 to DNA lesions, in turn controlling the recruitment of EXO1, which plays a role in regulating DNA end resection and DNA repair. Specific cancer-associated mutations in the OB-folds of BRCA2 disrupt the interaction with PAR and stop the recruitment of BRCA2 to DNA lesions, strongly implicating the role of OB-folds in DNA repair and cancer etiology [101,102,103]. Tumors with BRCA2 mutations often show loss of heterozygosity, though this is not universal. In a KRAS-driven pancreatic cancer mouse model, germline BRCA2 heterozygous mutation promoted tumor formation with no loss of wild-type BRCA2 allele. Therefore, this study suggests haploinsufficiency of BRCA2 genes and promotion of cell proliferation. Importantly, this conclusion does not take into consideration the role of epigenetics as a possible inhibitor of the wild-type BRCA2 [98].

Loss of BRCA2 function sensitizes cells to PARP inhibitors, which have been clinically exploited to treat an increasing number of cancers that harbor such mutations [104,105,106,107]. This synthetic lethal interaction provides the therapeutic window for treatment. However, while there are important and significant increases in progression free survival, time to progression and overall survival in many tumor types, cures are still rare [108,109]. The OB-folds of BRCA2 represent interesting targets that could inhibit BRCA2 function in wildtype tumors. The challenge, however, would be one of tumor specificity and a therapeutic window. However; if successful, the opportunities for expansion of the clinical utility of a PARP inhibitor into BRCA wildtype cancers would be significant.

## 7. Conclusions

The OB-fold motif has proven to be an effective module to endow ssDNA binding activity to proteins involved in genome stability and maintenance. Most proteins and complexes that employ OB-folds do so by tethering a number of independent modules together, either in a single polypeptide chain as seen in hSSB1/2 and BRCA2 or as part of multimeric complexes as seen in the MCM helicase. Other proteins employ both structural compositions including the ssDNA binding proteins RPA and CST. Targeting the interaction of the OB-fold with DNA has been successful in the case of RPA and the expansion to the other OB-fold proteins including CST, MCM, and others is primed for development. Targeting the vast array of OB-fold proteins with differing roles in genome maintenance via a single motif could be viewed as similar to targeting kinases 20 years ago. The perceived inability to drug such an interaction, challenges of specificity, solubility, and on- and off-target toxicity were all encountered and overcome such that there are over 50 FDA approved kinase inhibitors deployed in the clinic to combat cancer. Protein–DNA interactions catalyzed by OB-folds could therefore represent the next generation of innovative precision therapeutics for the treatment of cancer.

## Data Availability

Not applicable.
